# Case Report: Sequential treatment with rituximab and belimumab in a pediatric patient of type 1 diabetes mellitus complicated with systemic lupus erythematosus

**DOI:** 10.3389/fped.2025.1619143

**Published:** 2025-08-25

**Authors:** Fangfang Li, Yuci Zhang, Jing Yin, Linsheng Zhao, Chongwei Li

**Affiliations:** Department of Rheumatology and Immunology, Tianjin Children's Hospital (Children's Hospital, Tianjin University), Tianjin Key Laboratory of Birth Defects for Prevention and Treatment, Tianjin, China

**Keywords:** type 1 diabetes mellitus, systemic lupus erythematosus, diabetic kidney disease, lupus nephritis, rituximab, belimumab

## Abstract

Type 1 diabetes mellitus (T1DM) and systemic lupus erythematosus (SLE) are both autoimmune diseases influenced by multiple genetic and environmental factors, but rarely coexist. This case describes a 13-year-old girl with early onset of T1DM who was diagnosed with SLE 12 years later, highlighting diagnostic and therapeutic challenges, particularly in distinguishing kidney involvement and management without exacerbating hyperglycemia. The patient presented with edema of the eyelids and lower limbs. Urinalysis revealed hematuria and proteinuria. High-titer antinuclear antibody and anti-double-stranded DNA were detected. SLE was diagnosed clinically. As T1DM and SLE both cause kidney damage, kidney biopsy was performed. Deposition of various immune complexes led to a diagnosis of lupus nephritis. To avoid the impact of steroid pulses on glycemic control, conventional dose of steroids with sequential treatment with rituximab and belimumab was initiated. The combined therapy effectively alleviated the SLE condition, reduced steroids dosage, and led to discontinuation of steroids after 13 months. However, due to the prolonged disease course of T1DM, the pancreatic cell function was not reversed.

## Introduction

Type 1 diabetes mellitus (T1DM) and systemic lupus erythematosus (SLE) are both relatively prevalent in pediatric populations, though their co-occurrence is clinically uncommon. There were 5 cases of T1DM complicated with SLE reported (1–5) (Table 1), 2 of them were children. While the precise interaction mechanisms between T1DM and SLE remain unclear, substantial evidence indicates shared genetic risk factors between these conditions. Research has identified regulatory T cells (Tregs) as a distinct cellular lineage that plays a pivotal role in immune regulation. Treg dysfunction has been well-documented in various autoimmune disorders, including T1DM, SLE, rheumatoid arthritis, and others (6). Renal involvement frequently occurs in both T1DM and SLE, sharing overlapping clinical manifestations. Accurate identification of the underlying etiology of renal impairment is critical for appropriate therapeutic strategies. The morphological characteristics observed in renal biopsy play a crucial role in the diagnosis and classification of nephropathy (4). Glucocorticoids serve as a cornerstone in the treatment of SLE, though their potential impact on glycemic control requires careful monitoring. Currently, B-cell-targeted immunotherapy has been successfully implemented in clinical practice for SLE and is also under investigation for T1DM (7, 8). This case report presented a pediatric patient initially diagnosed with T1DM who subsequently developed SLE with lupus nephritis (LN). In this case, sequential treatment with rituximab and belimumab not only effectively alleviated SLE symptoms but also significantly mitigated the adverse effects of glucocorticoids on glycemic control.

**Table 1:** Clinical data of T1DM combined with SLE cases reported in the literature.

Case	Sex	Age of T1DM	Age of SLE	Lupus nephritis	Other manifestations	Treatment
		(year)	(year)			
1 (1)	Female	4	15	Yes	Arthritis	Oral methylprednisolone, Intravenous cyclophosphamide
2 (2)	Female	6	15	No	Celiac disease; systemic scleroderma	Prednisone (15 mg/day), tildiem (120 mg/day)
3 (3)	Female	14	21	No	Hydrohymenitis	Methyllprednisolone 80 mg/day, azathioprine
4 (4)	Female	13	28	Yes	Antiphospholipid syndrome	Glucocorticosteroid and cyclophosphamide pulses, mycophenolate, hydroxychloroquine,warfarin
5 (5)	Female	No mentioned	28	Lupus podocytopathy	Autoimmune hemolytic anemia	Methylprednisolone 250 mg/day for 4 days, mycophenolate, hydroxychloroquine

## CSAE description, laboratory test, diagnostic, therapeutic intervention, follow-up, and outcomes

The patient was diagnosed with T1DM during her first year of life. In September 2009, she was admitted to the department of Endocrinology with a 3-day history of polydipsia, polyuria, vomiting and diarrhea. Laboratory tests were fasting blood glucose >11. 1 mmol/L and hemoglobin A1c (HbA1c) of 10.3%. The presence of anti-islet cell antibodies and anti-glutamic acid decarboxylase antibodies confirmed the diagnosis of T1DM, and insulin therapy was initiated. After discharge, the patient failed to maintain regular follow-up visits, resulting in suboptimal glycemic control with blood glucose levels fluctuating between 2.2–20 mmol/L. Family history was notable for father diagnosed with type 2 diabetes mellitus in 2009, currently on combined therapy of oral metformin and subcutaneous insulin with good glycemic control.

The patient was rehospitalized at age 13 due to generalized edema in April 2022. Two weeks prior to admission, she developed periorbital and facial edema, which gradually progressed to involve the lower extremities. Five days before admission, urinalysis revealed hematuria and proteinuria, while complete blood count showed leukopenia. Throughout the disease course, she remained afebrile without rash or joint swelling and pain. Fasting blood glucose levels over the past week have fluctuated within the range of 7–8 mmol/L.

Laboratory test: Table 2.

**Table 2:** The laboratory test of our case.

Laboratory test	Result
CBC	WBC 2.40 × 109 cell/L, Hb 100 g/L, PLT 167 × 109 cell/L, Rec 2.36%
Urinalysis	PRO 2+, RBC 1+/HP
24-hour urine protein	953.4 mg/d (0–150 mg/d)
Urine cultures	Negative
Renal function	SCr 80 μmol/L (33–75 μmol/L), BUN 7.25 mmol/L (2.5–6.5 mmol/L)
Fasting blood glucose	7.07 mmol/L (3.90–6. 10 mmol/L)
HbA1c	7.5% (<6.5%)
Islet function	insulin <1.39 pmol/L (17.8–173 pmol/L), c-peptide <0.003 nmol/ml (0.370–1.470 nmol/ml)
ANA	1:640 (<1:80)
ENA	dsDNA 190.89 IU/ml (0–20 IU/ml), SSB 26.82 RU/ml (0–20 RU/ml), His 43.85 RU/ml (0–20 RU/ml), Nuc 226.06 RU/ml (0–20 RU/ml)
Coombs test	Positive
Immunoglobulin	Normal
Complement	C3 0.34 g/L (0.90–1.80 g/L), C4 0.02 g/L (0.10–0.40 g/L)
Whole-Exome Sequencing	Negative

CBC, complete blood bount; SCr, serum creatinine; BUN, blood urea nitrogen; HbA1c, hemoglobin A1c; ANA, antinuclear antibody; ENA, extractable nuclear antigens.

**Figure 1:**
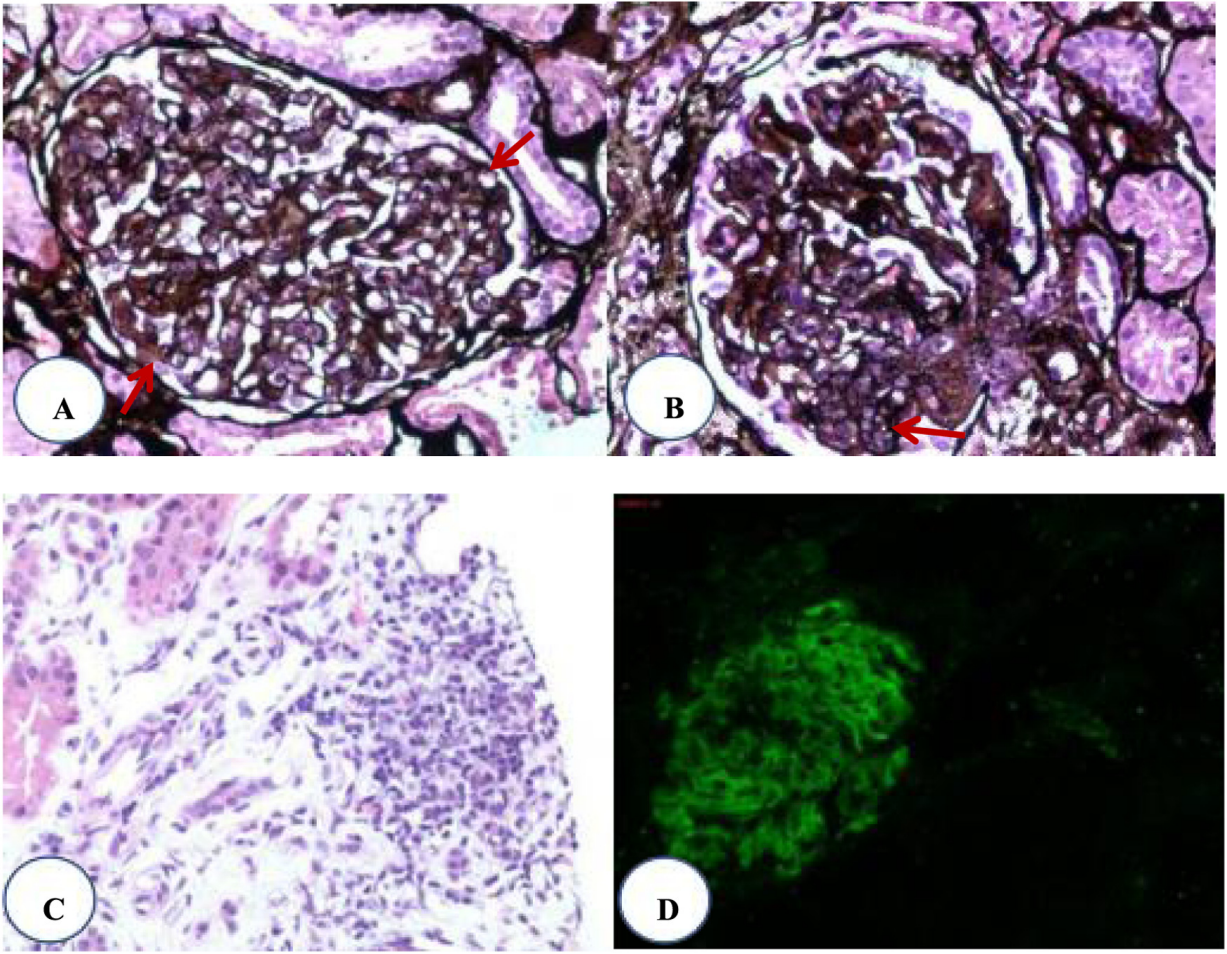
Renal pathology. **(A)** Diffuse proliferation of glomerular endothelial cells. **(B)** Segmental mesangial proliferation. **(C)** Focal infiltration of lymphocytes in the renal interstitium. **(D)** Immunofluorescence showing IgG deposition on the basement membrane.

Renal pathology: (Figure 1) 31 glomerulis were observed, one of them showed cellular crescents. The remaining glomerular endothelial cells showed diffuse hyperplasia (Figure 1A). Mesangial cells and matrix showed mild diffuse hyperplasia (Figure 1B). Interstitial focal lymphocyte and mononcyte infiltration accompanied by fibrosis were observed (Figure 1C). Immunofluorescence revealed IgG (++) (Figure 1D), IgA (++), IgM (++), C3 (++), C1q (+), Fn (±). Granular deposits were found in the mesangial region of the capillary loop. The final diagnosis was LN (class IVa + V). According to the scoring criteria of the Lupus Nephritis Activity Index and Chronicity Index, the Activity Index (AI) = 7 points (endocapillary hypercellularity 3 points + neutrophil infiltration and/or karyorrhexis 1 point + fibrinoid necrosis 0 points + subendothelial deposits 1 point + cellular crescents 1 point + interstitial cell infiltration 1 point), and the Chronicity Index (CI) = 0 points.

Diagnosis: The adolescent girl was diagnosed with T1DM at 1-year-old. She had renal involvement, hematological damage, positive antinuclear antibody and anti-double-stranded DNA antibody, decreased complement levels, and renal pathology consistent with LN.

According to the 2012 Systemic Lupus International Collaborating Clinics (SLICC) classification criteria, she was diagnosed with SLE.

**Figure 2:**
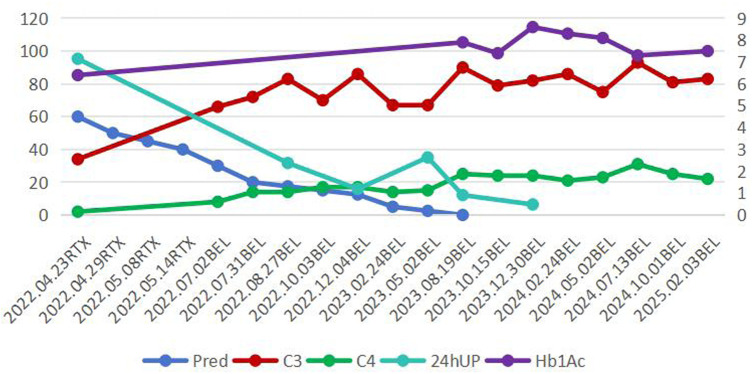
Treatment curve. Pred: Prednison (mg/d); C3, Complement C3 (mg/dl); C4, Complement C4 (mg/dl); 24hUP, Urinary Protein/24 h (cg/24 h); RTX, rituximab; BEL, belimumab; HbA1c, hemoglobin A1c (%) (right vertical axis).

Treatment, Follow-up and Outcomes (Figure 2): Due to the underlying T1DM, methylprednisolone pulse therapy was not used. She received oral prednisone 60 mg/day in combination with rituximab (RTX) (500 mg/w, a total of 4 doses). Oral prednisone was tapered to 50 mg, 40 mg, 35 mg, 30 mg, 25 mg, 20 mg, 17.5 mg, 15 mg, 12.5 mg, 10 mg, 7.5 mg, 5 mg, 2.5 mg at 2, 4, 6, 10, 12, 14, 18, 22, 30, 34, 38, 44, 52 weeks respectively, Belimumab (10 mg/kg × 4 doses, every 4 weeks; 10 mg/kg × 8 doses every 8 weeks; 10 mg/kg × 3 doses every 12 weeks) was subsequently initiated, combined with mycophenolate mofetil (MMF) (20 mg/kg.d) and hydroxychloroquine (HCQ) (4 mg/kg.d). In May 2023, corticosteroids were discontinued. By April 2025, the patient had received 15 doses of belimumab, MMF was tapered to 10 mg/kg.d, the dose of HCQ remain 4 mg/kg.d, with clinical symptom relief, negative urine protein, normal complement levels, and HbA1c level of 7%–7.5%. No serious infections and diabetes ketoacidosis occurred during treatment.

## Discussion

T1DM and SLE are both autoimmune diseases but rarely coexist. A study by Kota et al. (9) reported that 3 out of 260 T1DM patients were also diagnosed with SLE. Data from the Childhood Arthritis and Rheumatology Research Alliance (CARRA) in the United States revealed that among 388 pediatric SLE patients, only one had concurrent T1DM, indicating an extremely low prevalence of 0.26% (10). Children with SLE can produce various autoantibodies (e.g., islet cell antibodies, insulin antibodies), leading to pancreatic islet cell destruction.In this case, the child was initially diagnosed with T1DM and developed SLE 12 years later which is exceptionally rare.

The genetic susceptibility of both T1DM and SLE is strongly associated with the human leukocyte antigen (HLA) genes. Recent studies have identified that mutations in non-HLA genes such as *FOXP3, TNFAIP3, CTLA-4* and *PTPN22* may contribute to the co-occurrence of both conditions (11–13), suggesting potential shared pathogenic mechanisms, which regulate the function of Treg cells. Treg cell dysfunction has been closely linked to autoimmune pathogenesis, with impaired Treg function consistently observed in both T1DM and SLE patients. Notably, the transcription factor FOXP3 plays a pivotal role in Treg lineage commitment and functional maintenance. However, genetic testing in this case failed to identify any definitive disease-causing variants, the cause of her illness remained unclear.

Lupus nephritis (LN) represents the most prevalent and critical organ damage in pediatric SLE, serving as a key prognostic determinant. Diabetic kidney disease (DKD) occurs in approximately 50%–70% of children with T1DM, though it typically manifests only after more than 5 years of diabetes duration (14). In its early stages, DKD may present solely as microalbuminuria without overt symptoms. As the condition progresses, renal insufficiency may develop, ultimately leading to end-stage renal disease, the primary cause of mortality in pediatric T1DM patients. While both renal disorders share similar clinical presentations (with proteinuria being the most common feature), they exhibit distinct pathological characteristics. DKD typically demonstrates glomerular basement membrane thickening, progressing to diffuse or nodular mesangial expansion (forming “Kimmelstiel-Wilson nodules”) and glomerulosclerosis (15). These pathological changes are irreversible, with immunofluorescence typically showing negative results. In contrast, LN results from deposition of various immunecomplexes, and may develop cellular crescents during active phases. The renal pathological findings in this pediatric case were consistent with LN.

Long-term use of glucocorticoids can increase the risk of hyperglycemia and even induce ketoacidosis. Elevated blood glucose levels can further exacerbate kidney damage, posing challenges for the treatment in SLE patient complicated with diabetes. In the present case, biologic therapy was employed to reduce the use of steroids. The abnormal activation of B lymphocytes is a key factor in the pathogenesis of SLE (16), as B-cell activation leads to the production of large quantities of autoantibodies, resulting in multi-organ damage. RTX can directly deplete B cells and acts quickly, while belimumab specifically inhibits B lymphocyte stimulator, further suppressing B lymphocyte proliferation and differentiation (17). Sequential therapy with RTX and belimumab has been successfully applied in refractory SLE cases (18). Moreover, studies have shown that RTX in T1DM can temporary preserve pancreatic β-cell function and slow the decline in C-peptide levels, however, no significant improvement in long-term prognosis was observed (19). In this case, biologic therapy effectively alleviated SLE activity, reduced steroid dosage, and allowed timely tapering and discontinuation, thereby avoiding the increased T1DM burden associated with long-term steroid use, without diabetic ketoacidosis and severe infectious events occured. However, the pancreatic cells have already damaged due to the prolonged history of T1DM, the pancreatic cell function had not been reversed.

## Conclusion

In summary, the coexistence of T1DM and SLE is possible but clinically rare. Both diseases can lead to kidney damage, necessitating differentiation through renal biopsy. Sequential treatment with rituximab and belimumab can be successfully used in such cases while mitigate the impact of steroids on glycemic control.

## Data Availability

The original contributions presented in the study are included in the article/Supplementary Material, further inquiries can be directed to the corresponding author.
